# IRX5 promotes NF‐κB signalling to increase proliferation, migration and invasion via OPN in tongue squamous cell carcinoma

**DOI:** 10.1111/jcmm.13664

**Published:** 2018-05-15

**Authors:** Liyuan Huang, Fangfang Song, Hualing Sun, Lu Zhang, Cui Huang

**Affiliations:** ^1^ The State Key Laboratory Breeding Base of Basic Science of Stomatology (Hubei‐MOST) & Key Laboratory of Oral Biomedicine Ministry of Education (KLOBM) School & Hospital of Stomatology Wuhan University Wuhan Hubei China

**Keywords:** homeobox transcription factor, Iroquois homeobox gene 5, NF‐κB pathway, osteopontin, tongue squamous cell carcinoma

## Abstract

Iroquois homeobox gene 5 (*Irx5*) is a highly conserved member of the Iroquois homeobox gene family. Members of this family play distinct and overlapping roles in normal embryonic cell patterning and development of malignancies. In this study, we observed that IRX5 was abnormally abundant in tongue squamous cell carcinoma (TSCC) tissues and cell lines. We used gain‐ and loss‐of‐function methods to overexpress and knockdown IRX5 expression in the TSCC cell line CAL27. Our results elucidated that elevated levels of IRX5 promoted proliferation, migration and invasion of TSCC cells, whereas stable or transient knockdown of IRX5 expression suppressed TSCC cell proliferation, migration and invasion. As a transcription factor, IRX5 performed this function by targeting osteopontin (OPN) promoter and activating the NF‐κB pathway. Finally, studies in xenograft tumour model showed that IRX5 significantly enhanced OPN expression and promoted tumour growth. Taken together, our study elucidates a promotive effect of IRX5 in TSCC through the connection with OPN. These findings reveal the new molecular mechanism of TSCC, which may potentiate its use as a novel molecular therapy target for TSCC.

## 1 INTRODUCTION

Head and neck squamous cell carcinoma (HNSCC) is ranked sixth among the most common solid cancers worldwide,^1^ with approximately 686, 328 new cases per annum.^2^ Despite advanced surgical techniques in various combinations with radiation and chemotherapy, the overall 5‐year survival rate of HNSCC patients is <50%.^3^ Tongue squamous cell carcinoma (TSCC) is a common subtype of HNSCC, which is more biologically aggressive than other oral HNSCC cancers.^4^ Therapeutic target treatment for TSCC has not been currently met. Exploring the precise molecular mechanism and identifying a new therapeutic marker is crucial for the clinical diagnosis and treatment of TSCC.

As a member of the small integrin‐binding ligand N‐link glycoprotein (SIBLING) gene family,^5^ osteopontin (OPN) is an essential regulator of tumour progression, which was elevated in multiple human malignancies development.^5^, ^6^, ^7^ OPN plasma levels also act as a potential prognostic factor for tumour relapse and survival.^8^, ^9^ Gu et al reported that OPN knockdown decreased colorectal cancer metastasis both in vivo and in vitro.^10^ In HNSCC, the hypoxia signals significantly increased OPN expression, which enhanced cancer angiogenesis, growth and metastasis.^11^ Bioinformatics analysis indicates that the promoter of OPN gene contains potential transcriptional binding sites for Iroquois homeobox 5 (*Irx5*), suggesting that IRX5 might be a promising mediatory factor affecting OPN gene expression.

Irx genes encode a family of transcription factor with an extremely conserved homeobox domain and a 13 amino‐acid characteristic Iroquois domain.^12^ The Iroquois family contains 6 genes in mice and humans, which are organized into two cognate clusters, IrxA (Irx1, Irx2 and Irx4) and IrxB (Irx3, Irx5 and Irx6), and located in two different chromosomes.^13^ Irx genes play pivotal roles in cell specification and patterning during development processes, as well as human carcinomas, either as activators or suppressors;^12^, ^14^ this dual effect differs in different Irx gene and species. Notably, IRX5 is enhanced in colorectal cancer and human adenomas,^15^, ^16^ and IRX5 silencing inhibited LNCaP prostate cancer cell apoptosis.^17^ However, although several studies regarding the connection of IRX5 with cancer have been reported, the accurate functions of IRX5 in the progression and suppression of TSCC remains largely indistinct. Therefore, the aim of this study was to demonstrate the relationship of IRX5 with TSCC and to examine the potential novel target of IRX5.

In this study, we found that IRX5 was significantly up‐regulated in TSCC tissues and cell lines. The expression of IRX5 is associated with TSCC cell proliferation, migration and invasion. Moreover, IRX5 exerts this role through targeting OPN promoter, and NF‐κB pathway also participated in this promoting progress.

## 2 MATERIALS AND METHODS

### 2.1 Ethics approval and consent to participate

All of our experiments were approved by the Institutional Ethical Board of Wuhan University (Hubei, China) and were performed according to the guidelines of the National Institute of Health (NIH). Informed consent was obtained from all patients. The protocol of all animal procedures was approved by the Committee of the Ethics of Animal Experiments of the Wuhan University of Stomatology.

### 2.2 Human tissue specimens

A total of 12 samples of human TSCC tissues and corresponding adjacent normal tongue tissues were used in this study. All patients, from whom the tissue samples were collected, were clinically and histopathologically diagnosed with TSCC at the School and Hospital of Stomatology, Wuhan University, and underwent surgical resection with the approval of the Ethics Committee. The formalin‐fixed, paraffin‐embedded portions of surgical specimens were subjected to IHC analysis, and the remaining portions were immediately frozen in liquid nitrogen and stored at −80°C in our laboratory. TSCC was histopathologically confirmed, after which the tumour specimens were used to extract mRNA and protein for qRT‐PCR and Western blot analysis.

### 2.3 Cell culture and reagents

Human cell lines (SCC9, SCC25 and CAL27) were purchased from the American Type Culture Collection (ATCC, Manassas, USA) and grown in DMEM (HyClone, South Logan, UT, USA) supplemented with 10% foetal bovine serum (FBS, Gibco, Grand Island, NY, USA), 100 U/mL of penicillin and 100 U/mL of streptomycin. All cell lines were grown at 37°C in 5% CO_2_ and air‐humidified incubator. Puromycin dihydrochloride (MDBio, Qingdao, China) was added at 2 μg/mL to screen transfected cells. BAY 11‐7082 (Beyotime Biotechnology, Shanghai, China) was used at 3 μmol/L to inhibit the NF‐κB signalling pathway (1 mmol/L stock in DMSO).

### 2.4 Human keratinocytes isolation and culture

Human keratinocytes were isolated from tongue noncancerous disease patients (16‐35 years old). Informed consent was obtained from all donors. Human tongue epithelial tissues were harvested and rinsed thrice in sterile phosphate buffered saline containing 2% antibiotics (100 U/mL, penicillin and 100 U/mL, streptomycin). The samples were separated from the underlying muscle or connective tissue, minced into pieces, and then incubated overnight at 4°C in Dispase II (Roche, Basel, Switzerland). The epidermal layer was separated from other tissues and treated with trypsin‐EDTA (Invitrogen, Carlsbad, CA, USA) for 15 minutes at 37°C to isolate KCs. Isolated KCs were then cultured in Keratinocyte Growth Medium (Promocell) at 37°C in 5% CO_2_ and air‐humidified incubator.

### 2.5 qRT‐PCR

Total RNA was extracted from indicated cells and tissue samples using TRIzol (Invitrogen). RNA at 1 μg was reverse transcribed into cDNA template using the PrimeScript™ RT Reagent Kit with gDNA Eraser (TaKaRa, Tokyo, Japan). qRT‐PCR was performed using the SYBR Premix Ex Taq II (Tli RNaseH Plus, TaKaRa) in the QuantStudio™ 6 Flex QPCR System (Applied Biosystems, Singapore). Primer sequences are listed in Table S1. All experiments were performed in triplicates.

### 2.6 Western blot analysis

CAL27 cells and tissue specimens were lysed on ice using RIPA buffer (Beyotime, ShangHai, China) containing PMSF (Roche) and phosphatase inhibitors (Roche) and centrifuged at 13,523 *g* for 10 minutes at 4°C to remove cell debris. Protein concentration was then measured using the BCA Assay kit (Biosharp, Hefei, China). Total protein concentrations in all samples were equalled, following which the proteins were denatured by heating at 95°C for 8 minutes. Nuclear proteins were extracted using the Nuclear and Cytoplasmic Protein Extraction Kit (Beyotime) following the manufacturer's instructions. A quantity of 30 mg protein was electrophoresed on 10%‐12% SDS‐polyacrylamide gel and transferred onto PVDF membrane (Millipore, Billerica, MA, USA). Membranes were blocked with 5% skim milk for 1 hour at room temperature, following which they were incubated with primary mouse monoclonal antibodies against human IRX5 (1:200; Sigma‐Aldrich, St. Louis, MO, USA), human OPN (1:500; Santa Cruz, CA, USA), IκBa (1:1000; Cell Signaling Technology, CA, USA), H3 (1:5000; ABclonal Technology, Oxfordshire, UK), β‐actin (1:5000; bioPM, China) and GAPDH (1:5000; bioPM) and primary rabbit polyclonal antibodies to human phosphorylated‐p65 S536 (1:1000; Cell Signaling Technology), NF‐κB‐p65 (1:1000; Cell Signaling Technology) and MMP2 (1:1000; ABclonal) on shaker at 4°C overnight. After 3 washes by TBST, the membranes were incubated with HRP‐conjugated secondary antibody at 37°C for 1 hour. Protein bands were visualized using ECL reagent. Then data were quantified by densitometry using Image J software (National Institutes of Health, Bethesda, Maryland, USA). Western blot analyses were repeated at least thrice.

### 2.7 Lentiviral transduction

Human full‐length of IRX5 CDS was cloned into an empty pLVX‐IRES‐puro (Clontech, Mountain View, CA, USA) plasmid and the V5 tag was added in Wuhan Miaoling Bioscience & Technology Co., Ltd (Wuhan, China). The construct was sequenced for verification and named PLVX‐IRX5‐puro‐V5. IRX5 was overexpressed using PLVX‐IRX5‐puro‐V5 plasmid named IRX5 and the empty pLVX‐IRES‐puro was used as control named Vector. IRX5 was inhibited using shRNA targeting the human IRX5 gene (Genechem, IRX5#1 AAAGACTCTCCCTATGAAT, IRX5#2 AAGGTATGTCCGACATTTA). Nonsense shRNA hU6‐MCS‐Ubiquitin‐EGFP‐IRES‐puromycin (Genechem, shNC, TTCTCCGAACGTGTCACGT) was used as negative control. 2.5 × 10^6^ 293E cells were plated onto a 6 cm dish. Three plasmid systems, including pMD2.G and psPAX2, were co‐transfected according to the manufacturer's instructions of TurboFect (TurboFect, Thermo Fisher, USA). After 48 hours, the lentiviral supernatant was collected, centrifuged and filtered through a 0.22 μm filter. For infection, CAL27 cells were incubated with lentivirals for 48 hours containing 5 μg/mL polybrene. The cells were named IRX5, Vector, shIRX5#1, shIRX5#2 and shNC. After 48 hours, all cells were screened by puromycin, and IRX5 expression was quantified using qRT‐PCR and Western blot analysis.

### 2.8 Transient transfection

siRNAs targeted IRX5 and OPN were synthesized from GenePharma (Suzhou, China). The sequences are listed in Table S2. Cells were transfected with targeting siRNAs or plasmids using Lipofectamine 2000 (Invitrogen) according to the manufacturer's instructions. IRX5 and OPN expressions were measured using qRT‐PCR and Western blot analysis.

### 2.9 Bioinformatics analyses

Osteopontin candidate promoter sequences from −2000 to +200 were retrieved from NCBI, and analysed using MatInspector software (http://www.genomatix.de/matinspector.html.) for the putative IRX5 binding site.

### 2.10 Dual luciferase assay

Putative OPN promoter fragment were amplified and the products were cloned into pGL3‐basic vector. The primer sequences of OPN promoter were listed in Table S1. CAL27 cells were plated onto 24‐well plate at a density of 1 × 10^5^ cells/well. Lipofectamine 2000 (Invitrogen) was co‐transfected with 0.5 μg of reporter plasmids, namely pGL3‐basic, OPN‐promoter or NF‐κB‐luc and 0.05 μg of the internal control plasmid pRL‐TK (Promega, Madison, WI, USA). Cells were lysed using passive lysis buffer (Promega) for 15 minutes according to the manufacturer's instructions. Firefly and Renilla luciferase activities were assessed using GloMax 20/20 Luminometer (Promega). The relative OPN or NF‐κB transcriptional activity (relative light units of firefly luciferase/Renilla luciferase, fRLU/rRLU) was counted.

### 2.11 CCK8 assay

Cell suspensions (100 μL) containing 5000 cells were plated on 96‐well plate per well. After incubation for the indicated time periods (24, 48, 72 and 96 hours), media were removed and replaced with 100 μL culture media containing 10 μL of CCK8 solution (Beyotime) for 2 hours. The supernatant was collected, and absorbance at 450 nm was measured using a microplate reader to calculate cell growth rate.

### 2.12 Wound healing assay

CAL27 cells were plated on 6‐well plate at a density of 3 × 10^5^ cells per well. When the cells reached 95% confluency, a sterile 20 μL pipette tip was used to make a wound scratch, and PBS was used to remove the detached cells. Phase contrast images were collected in the same field at indicated time periods (0, 24, 36 and 48 hours) using an inverted microscope (Leica).

### 2.13 Transwell assay

Cell migration and invasion assays were conducted using 24‐well transwells (8.0‐μm pore size) with one‐fourth dilution or without matrigel coating (BD, Franklin Lakes, NJ, USA). In total, 1‐3 × 10^5^ cultured cells in 200 μL of serum‐free DMEM (HyClone) medium were seeded into the upper Boyden chamber and 600 μL of the same medium containing 10% FBS (Gibco) was added to the lower chamber. After 24 hours of incubation, cotton swabs were used to remove the cells in the upper chamber, and the filters were then fixed with 4% paraformaldehyde for 15 minutes and washed with PBS. Finally, cells in the filter were stained with crystal violet and examined by microscope (Olympus). Migrating or invading cells in 5 random optical fields from triplicate filters were quantified and averaged.

### 2.14 Xenograft tumour model

Four‐week‐old female athymic BALB/c nude mice were purchased from the Hunan SJA Laboratory Animal Co. Ltd (Hunan, China). All 10 mice were maintained under specific pathogen‐free (SPF) conditions and divided into IRX5, Vector, shIRX5#2 and shNC groups (n = 5 per group). Tumour cells were inoculated into mice by subcutaneous injection of 200 μL of PBS containing 7 × 10^6^ cells into both sides of the upper back. Tumour growth was examined every 3 days and tumour volumes were calculated using the equation $\nu =\frac{1}{2}ab^{2}$ (ν, volume; *a*, longitudinal diameter; *b*, latitudinal diameter). After 5 weeks, mice were sacrificed and decapitated to compare the tumour weights and volumes. The tumours were then used for IHC analysis following the previous procedures. The protocol was approved by the Committee of the Ethics of Animal Experiments of the Wuhan University of Stomatology.

### 2.15 H&E staining and IHC

Paraffin‐embedded TSCC tissues and xenograft tumours were sectioned at 5 μm and mounted onto silane‐coated slides (Thermo Fisher, Waltham, MA, USA), and the sections were de‐waxed in xylene and rehydrated in graded alcohols. H&E staining and IHC were conducted on consecutive tissue sections respectively. IHC was performed according to the manufacturer's protocol (ZhongShan Biotech, Beijing, China). Sections were treated with the EDTA Antigen Retrieval Solution (Beyotime) at 95°C for 15 minutes. The slides were incubated in 3% hydrogen peroxide. Sections were blocked using 5% goat serum and then incubated with primary mouse polyclonal antibody to IRX5 (1:50; Santa Cruz, Texas, CA, USA) and mouse monoclonal antibodies to OPN (1:50; Santa Cruz) and Ki67 (1:100; ZhongShan Biotech) at 4°C overnight. Slides were then incubated with secondary goat anti‐mouse antibody for 15 minutes at 37°C. Diaminobenzidine–HCl (DAB, MXB, Fuzhou, China) was used as a substrate for visualization. Finally, haematoxylin was used to counterstain the nuclei.

### 2.16 Statistical analysis

All the quantitative parameters were given as mean ± SEM. Comparisons were analysed by Student's *t* test for 2 groups and ANOVA for more than 2 groups using SPSS version 17.0. *P* values <.05 were considered significantly different.

## 3 RESULTS

### 3.1 IRX5 is up‐regulated in TSCC tissues and cell lines

**Figure 1:**
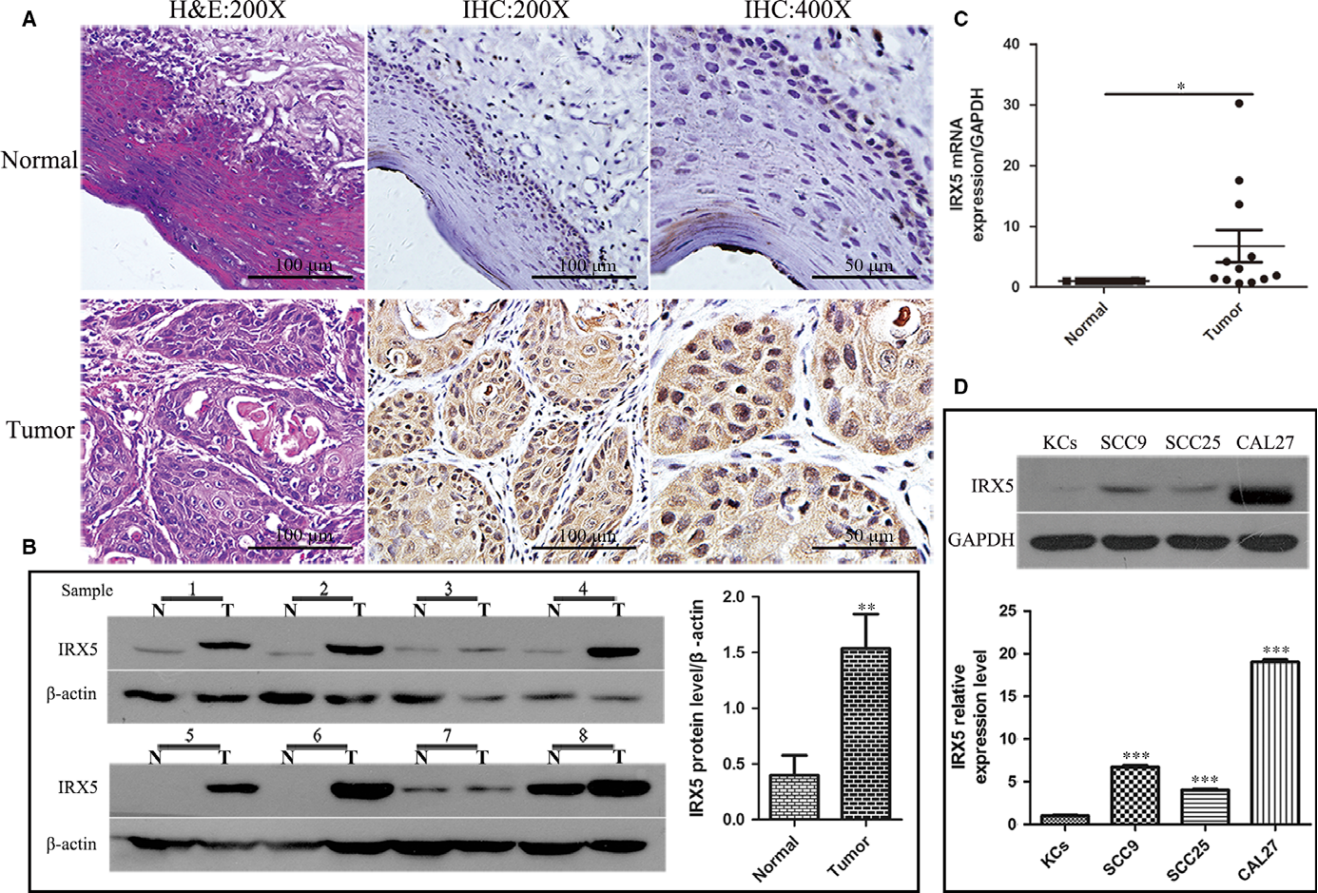
Iroquois homeobox gene 5 (IRX5) was up‐regulated in tongue squamous cell carcinoma (TSCC) tissues and cell lines. A, H&E and immunohistochemical analysis of IRX5 protein expression in TSCC and adjacent normal tongue tissue specimens. B, Western blot analysis of the IRX5 expression in 8 pairs of TSCC (T) and matched normal tongue tissue (N) specimens. IRX5 protein expression levels were normalized to β‐actin expression level. (n = 8). C, The mRNA level of IRX5 in TSCC samples and normal tongue tissues (n = 12). D, Western blot analyses expression levels of IRX5 in human KCs and 3 TSCC cell lines (SCC9, SCC25, CAL27). *P <* *.05, **P <* *.01, ***P <* *.001. Error bars represent the mean ± SD values

We first assayed IRX5 expression pattern in 12 pairs of human TSCC tissues and relevant adjacent normal tongue tissues. Immunological Histological Chemistry (IHC) analysis demonstrated that IRX5 levels were noticeable higher in TSCC than that in normal tissues (Figure 1A). Moreover, IRX5 protein and mRNA levels were also elevated in TSCC than in normal tissues, evidenced by Western blot and qRT‐PCR (Figure 1B,C). We then evaluated IRX5 protein levels of 3 human TSCC cell lines (SCC9, SCC25 and CAL27) and human normal tongue keratinocytes (KCs). Consistently, IRX5 protein levels were significantly higher in the 3 TSCC cell lines than in normal KCs (Figure 1D), and the expression of IRX5 was highest in CAL27. These results indicated that up‐regulated IRX5 expression might be involved in TSCC progression.

### 3.2 IRX5 overexpression promotes in vitro TSCC cell proliferation, migration and invasion

**Figure 2:**
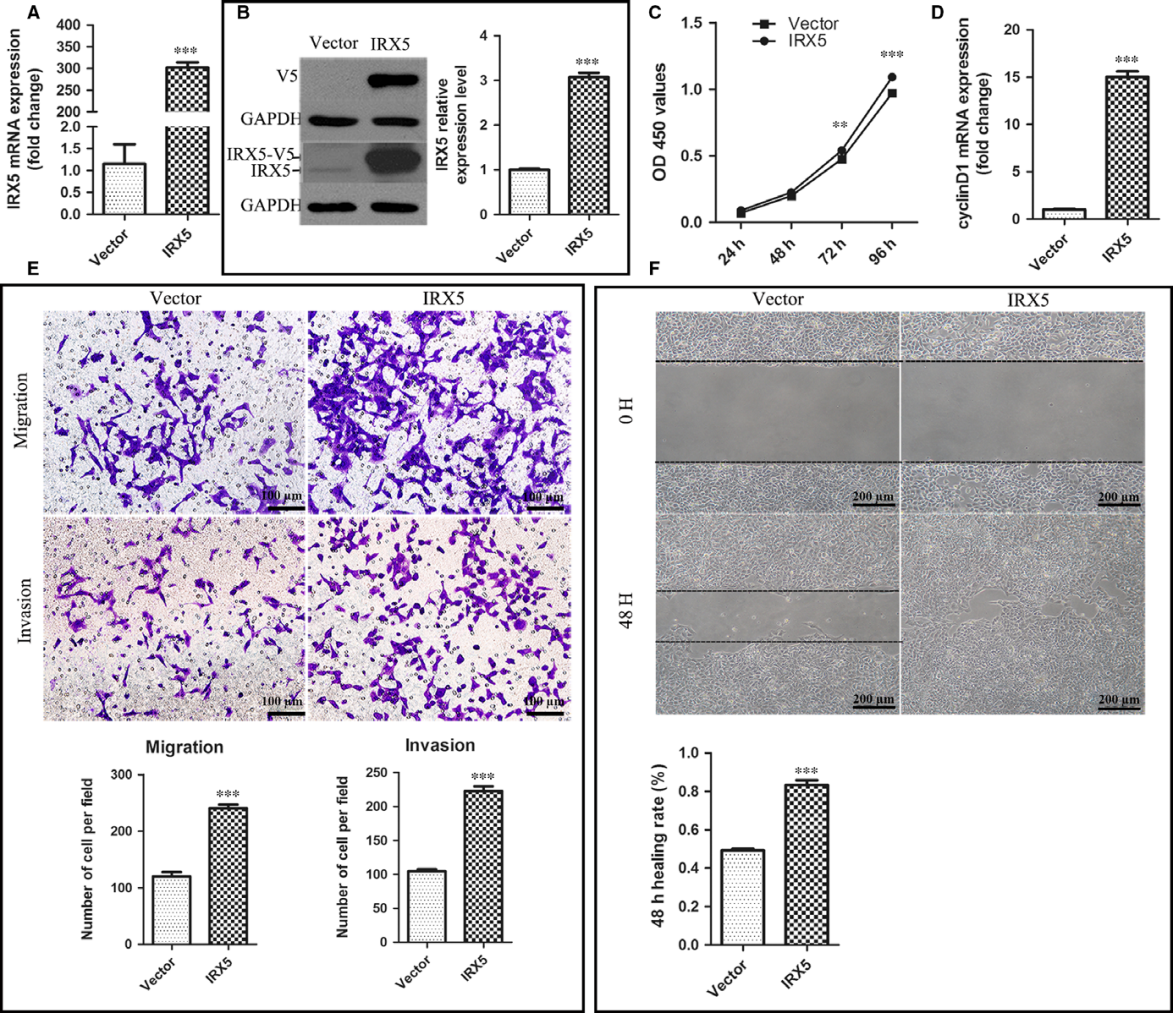
Iroquois homeobox gene 5 (IRX5) overexpression promotes in vitro tongue squamous cell carcinoma (TSCC) cell proliferation, migration and invasion. A, qRT‐PCR analysis of IRX5 mRNA level in CAL27 cell transfected with IRX5 (IRX5) and empty vector (Vector). B, Western blot analysis of IRX5 and V5 levels in CAL27 cell transfected with IRX5 (IRX5) and empty vector (Vector). C, CCK8 assay analysis of proliferation rate of CAL27 cell transfected with IRX5 (IRX5) and empty vector (Vector). D, qRT‐PCR analysis of cyclin D1 mRNA level. E, Overexpression of IRX5 resulted in an increase in the migratory and invasive abilities of CAL27 cell as measured by Transwell assay. F, IRX5 overexpression enhanced the migratory ability of CAL27 cell in a wound‐healing assay. **P <* *.01, ***P <* *.001. Error bars represent the mean ± SD values

To reveal whether IRX5 is involved in TSCC progression, we stably overexpressed exogenous IRX5 in CAL27 cells using lentiviral transduction system. IRX5 expression was quantitated by qRT‐PCR and Western blot analyses 48 hours post‐infection. IRX5 mRNA and protein levels were enhanced about 300‐ and 3‐fold respectively (Figure 2A,B). The CCK8 assay revealed that IRX5 overexpression markedly elevated proliferation rate of CAL27 cells (Figure 2C). mRNA levels of the G1 cell cycle control protein (cyclinD1), which has been demonstrated to promote tumorigenicity and growth of HNSCC in vitro and in vivo,^18^ were also increased relative to those in control (Figure 2D). Furthermore, well‐established metastasis‐associated wound healing and transwell assays suggested that IRX5 overexpression facilitates migration and invasion capacities of TSCC cells in vitro (Figure 2E,F).

### 3.3 IRX5 knockdown reduces TSCC cell growth, migration and invasion in vitro

**Figure 3:**
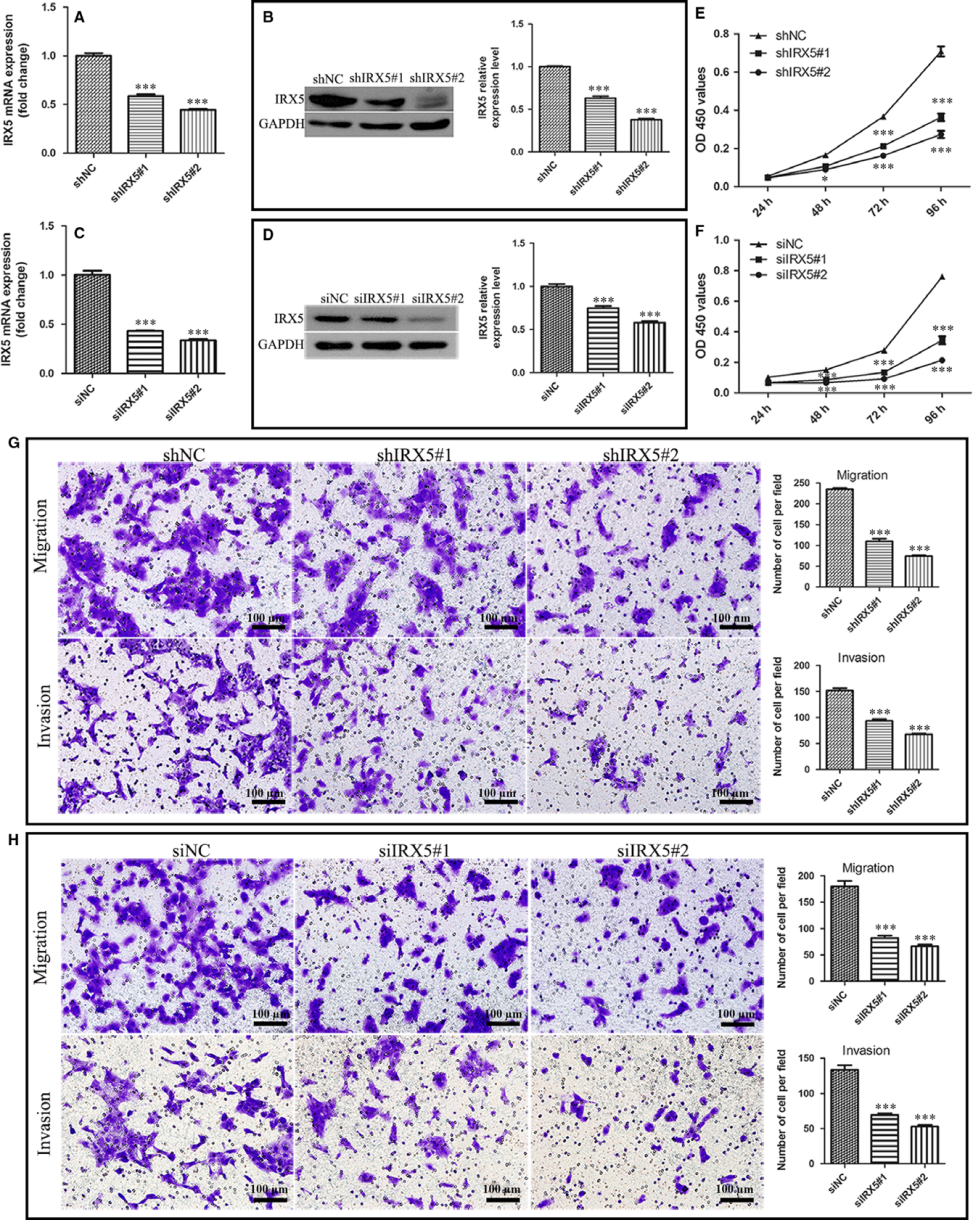
Iroquois homeobox gene 5 (IRX5) down‐regulation reduces tongue squamous cell carcinoma (TSCC) cell growth, migration and invasion in vitro. A, qRT‐PCR analysis of IRX5 mRNA level in CAL27 cell transfected with shIRX5#1 (shIRX5#1), shIRX5#2 (shIRX5#2) and negative empty vector (shNC). B, Western blot analysis of IRX5 protein expression level. C, qRT‐PCR showed that IRX5 mRNA level was decreased in CAL27 cell using RNAi (siIRX5#1, siIRX5#2) compared with a nonsense control (siNC). D, IRX5 expression level was reduced in siIRX5#1 and siIRX5#2. E, F, Knockdown IRX5 decreased proliferation rate of CAL27 cell. G, H, Both shRNAs and siRNAs against IRX5 inhibited the migration and invasion of CAL27 cell. *P <* *.05, ***P <* *.001. Error bars represent the mean ± SD values

Given increased IRX5 expression in TSCC cells, the loss‐of‐function studies were conducted using CAL27 cells either by stable transduction with two different short hairpin RNAs targeting IRX5 (shIRX5#1, shIRX5#2), or by transient transfection with two different short interfering RNAs (siIRX5#1, siIRX5#2). Cells infected with hU6‐MCS Ubiquitin‐EGFP‐IRES‐puromycin and siRNA negative control were designated as shNC and siNC respectively. IRX5 mRNA and protein levels were significantly decreased in TSCC cells than those in control cells (Figure 3A‐D). As expected, both shRNAs and siRNAs used for the knockdown of IRX5 expression led to a decreased proliferation rate of CAL27 cells compared with control cells (Figure 3E,F). In addition, depletion of IRX5 protein also reduced the ability of CAL27 cells to migrate through transwell membranes and Matrigel (Figure 3G,H).

### 3.4 OPN is a target of IRX5

**Figure 4:**
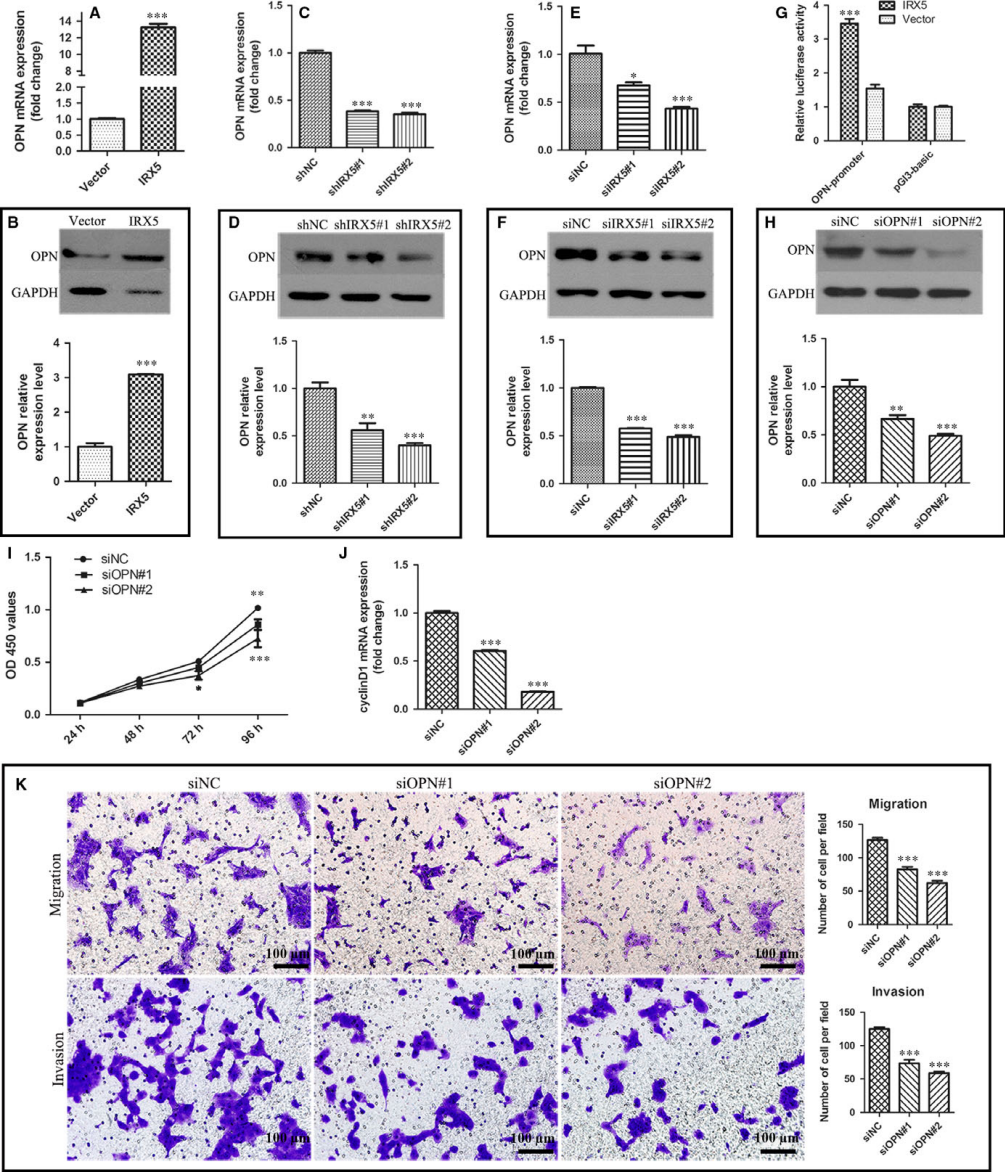
Osteopontin (OPN) is a target of Iroquois homeobox gene 5 (IRX5). A, mRNA level of OPN in IRX5 overexpressed CAL27 cell. B, Protein level of OPN in IRX5 overexpressed CAL27 cell. C, E, mRNA level of OPN in IRX5 down‐regulated CAL27 cell. D, F, Protein level of OPN in IRX5 down‐regulated CAL27 cell. G, OPN relative luciferase activity. OPN‐promoter: nucleotides, nts −2284 to −378 constructed into pGL3‐basic. Overexpression of IRX5 level elevated OPN promoter luciferase activity. H, Western blot analysis of OPN protein level down‐regulated by siRNA against OPN (siOPN#1, siOPN#2) compared with a nonsense control (siNC) in IRX5 overexpressed CAL27 cell. I, Down‐regulated OPN resulted in decreased proliferation rate of CAL27/IRX5 cell. J, mRNA level of cyclinD1 was decreased in CAL27/IRX5 cell when down‐regulating OPN expression. K, Transwell assay analysis of the ability of migration and invasion of IRX5 overexpressed CAL27 cell blocked by siOPNs. *P <* *.05, **P <* *.01, ***P <* *.001. Error bars represent the mean ± SD values

First, we detected OPN expression with respect to various levels of IRX5 expression. Results revealed that overexpressed IRX5 led to elevated OPN mRNA and protein levels (Figure 4A,B). Similarly, the knockdown of IRX5 expression concomitantly decreased OPN levels (Figure 4C‐F). Next, we analysed the OPN promoter for potential IRX5 binding sites. Using Genomatix MatInspector, we found that the human candidate OPN promoter sequence from −2000 to +200 contained one IRX5 binding sites. Furthermore, we hand‐screened the OPN promoter sequence from 3 mammalian species for the IRX5 binding sites (ACANNTGT). All OPN promoters contained the IRX5 binding sites (Table S1). To confirm that IRX5 directly regulates OPN promoter, dual‐luciferase reporter assay was performed. We cloned fragments of the human OPN promoter (between −2284 to −378) to pGL3‐basic luciferase plasmid, pGL3‐basic luciferase vector was used as control. And then we transfected these two plasmids respectively into the IRX5‐overexpressing CAL27 or IRX5‐Vector CAL27 cells. As demonstrated in Figure 4G, IRX5 overexpression markedly enhanced the OPN promoter activity. We then examined whether IRX5 promoted TSCC proliferation, migration and invasion by activating OPN. We used two OPN‐targeting siRNAs (siOPN#1, siOPN#2) for the knockdown of OPN expression in CAL27‐IRX5 cells, siNC as control (Figure 4H). The CCK8 assay and cyclinD1 expression demonstrated that transfection with siOPNs resulted in a dramatically decreased proliferation rate of CAL27‐IRX5 cells (Figure 4I,J). Similarly, the transwell assay demonstrated that siOPN rescued the migration and invasion capacities of CAL27‐IRX5 cells (Figure 4K). These results suggest that IRX5, at least partially, promotes TSCC cell proliferation, migration and invasion by targeting OPN.

### 3.5 NF‐κB pathway activation is involved in IRX5/OPN‐induced TSCC proliferation, migration and invasion

**Figure 5:**
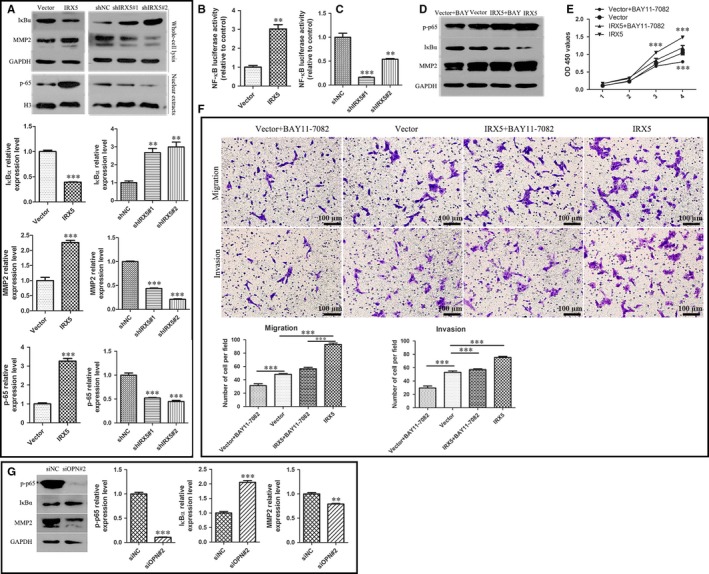
NF‐κB pathway activation is involved in Iroquois homeobox gene 5 (IRX5)/osteopontin (OPN)‐induced tongue squamous cell carcinoma (TSCC) proliferation, migration and invasion. A, Western blot analysis of NF‐κB relative protein and MMP2 protein level in IRX5, Vector, and in shIRX5#1, shIRX5#2, shNC CAL27 cells. B, C, NF‐κB luciferase activity was detected in indicated cells above. D, IRX5 and Vector CAL27 cells were treated with 3uM BAY11‐7082 respectively (IRX5 + BAY, Vector+BAY), NF‐κB relative protein and MMP2 protein level were measured in 4 groups cells. E, CCK8 assay analysis of proliferation rate of 4 groups. F, Transwell assay demonstrated that BAY11‐7082 decreased migratory and invasive ability in IRX5 and Vector cells. G, Western blot analysis of p‐p65, IκBα, MMP2 expression level in IRX5 overexpressed CAL27 cell transient with siOPN#2 or siNC. **P <* *.01, ***P <* *.001. Error bars represent the mean ± SD values

It has been reported that OPN modulates the NF‐κB pathway in various cancers.^19^, ^20^ Therefore, we examined whether IRX5 acted as an upstream of OPN/NF‐κB. Our results indicated that IRX5 overexpression resulted in increased nuclear p65 level and led to increased IκBα degradation. Furthermore, levels of MMP2, which acts as the downstream of the NF‐κB pathway,^21^ were also elevated in IRX5 overexpressing cells. Consistent with these results, the knockdown of IRX5 expression using shRNA resulted in decreased nuclear p65, MMP2 expression and IκBα degradation (Figure 5A). In addition, IRX5 overexpression boosted NF‐κB luciferase activity (Figure 5B). Conversely, the knockdown of IRX5 expression suppressed NF‐κB luciferase activity (Figure 5C). BAY11‐7082, a NF‐κB signalling pathway inhibitor,^22^ decreased IκBα phosphorylation and degradation to block the NF‐κB pathway activity (Figure 5D). Our results revealed that BAY11‐7082 treatment inhibited the proliferation rate of CAL27 cells transfected with either IRX5 or an empty vector (Figure 5E). Similarly, BAY 11‐7082 stimulation attenuated migration‐promoting effect of CAL27‐IRX5 cells (Figure 5F). We then examined the levels of phosphorylated p65 and MMP2 as well as IκBα degradation in CAL27‐IRX5 cells treated with siOPN#2, and we observed that the previously elevated expression and degradation of the corresponding factors were decreased (Figure 5G). These findings suggest that IRX5 regulates TSCC cell proliferation, migration and invasion by promoting the OPN/NF‐κB pathway.

### 3.6 IRX5 promotes TSCC progression in vivo

**Figure 6:**
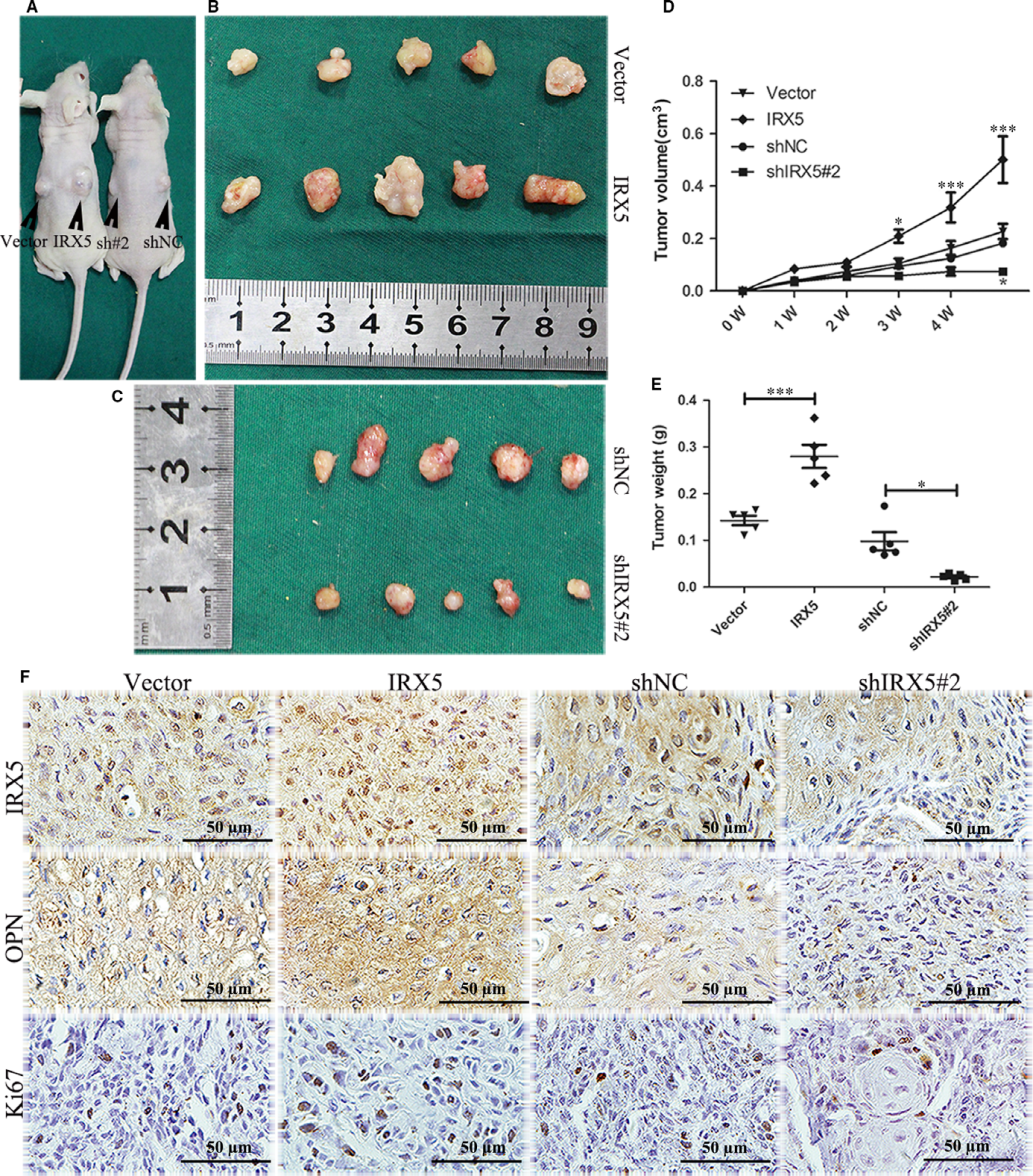
Iroquois homeobox gene 5 (IRX5) promotes tongue squamous cell carcinoma (TSCC) progression in vivo. A, Two representative mice of xenograft tumour in vivo. B, C, Tumours were stripped out of mice in all groups. D, Tumour volumes were measured from 0 W to 5 W of 4 groups. E, Tumour weight of all groups. F, IHC analysis of the expression of IRX5, osteopontin (OPN) and Ki67 in each groups of mice tumours. *P <* *.05, ***P <* *.001. Error bars represent the mean ± SD values

We finally examined the biological effect of IRX5 on TSCC tumorigenesis in a xenograft mouse model. CAL27 cells transfected with IRX5, empty vector, shIRX5#2, or shNC were subcutaneously injected into nude mice, and tumour volume was recorded every 3 days. Two mice represented tumours from each of the 4 groups were shown in Figure 6A. A significantly increase in the tumour volume and weight was observed in IRX5‐overexpressing group (Figure 6B‐E). After 5 weeks of xenograft growth, immunohistochemical staining revealed that IRX5‐overexpressing groups showed higher IRX5 expression compared to tumours from the control group (Figure 6F). Likewise, tumours from the IRX5‐overexpressing group displayed increased OPN expression. On the contrary, the results revealed the presence of lower levels of IRX5 and OPN in IRX5‐knockdown group compared with the control group. The tumour cells proliferation was increased in tumours from the IRX5‐overexpressing groups and decreased in IRX5‐knockdown groups, as evidenced by the staining density of Ki‐67 positive cells (Figure 6F).

## 4 DISCUSSION

Recently, dysregulation of Irx genes has been observed to have functional significance for multiple cancers, which has been reported to play either a promotive role or a repressive role in regulating tumour progression and metastasis.^23^, ^24^ Better knowledge of cancer‐related transcription factors is critical for gaining novel mechanical insights into carcinogenesis and clarifying effective therapies for cancers. In normal tissues, IRX5 is involved in various cellular activities, such as cell proliferation, cell specification and vascular remodelling.^25^ Meanwhile, these processes may contribute to both cancer progression and maintaining tumour microenvironment during tumour growth and metastasis. However, the association of dysregulated IRX5 and TSCC, and their expression pattern and underlying molecular mechanisms in TSCC cells are poorly understood.

In our study, we observed that IRX5 was up‐regulated in TSCC tissues compared with adjacent normal tongue tissues. The aberrant IRX5 expression indicates that IRX5 might participate in TSCC progression. IRX5 was also elevated in TSCC cell lines with CAL27 cells exhibiting the highest expression. Accordingly, this cell line was chosen for subsequent analysis. Moreover, IRX5 accelerated CAL27 cell proliferation, migration and invasion in vitro and in vivo. To the best of our knowledge, this is the first study that examined the role of IRX5 in TSCC progression. In accordance with our study, it has been reported that IRX5 play crucial effects in various tumour types. For example, it was reported that IRX5 may regulate LNCaP prostate cancer cells apoptosis partially through p53 and cells cycle by mediating p21 levels^17^; In another study, IRX5 transcriptionally suppresses the Dpp/TGF‐β pathway in human adenomas, which excessively activates the Wnt and EGFR/Ras signalling pathways, thus conferring a growth advantage to tumour cells.^16^ This prompts us that IRX5 may be exploited as a promising therapeutic target for the treatment of TSCC.^15^, ^16^, ^17^

Here we delineated a new regulatory pathway in which IRX5 transcriptionally regulates OPN and thus, impacts cancer progression. OPN plays a crucial tumour‐promotive effect in the occurrence and progression of different cancers. In several types of cancers, the levels of OPN, including the plasma level of OPN, were significantly increased, such as pancreatic ductal adenocarcinoma,^26^ prostate cancer.^27^ Moreover, plasma levels of OPN has been demonstrated to act as potential prognostic factors for tumour relapse and survival.^28^ In HNSCC, OPN enhances tumour angiogenesis, growth and metastasis.^11^ Irx genes have been reported to recognize and bind to a palindromic sequence ACANNTGT to transcriptionally regulate target genes.^29^ Using MatInspector, we found that there are IRX5 transcriptional binding sites in the OPN promoter. We then searched 3 OPN promoters, pig, rat, cattle, and detected the presence of ACANNTGT motifs. Based on the above‐mentioned that the OPN promoter contains IRX5 transcriptional recognition motifs, we investigated whether IRX5 transcriptionally interacts with OPN and enhances biological characteristics of TSCC. In our study, OPN expression was positively correlated with IRX5 levels in vitro and in vivo. The luciferase assay revealed that OPN promoter activity was enhanced by IRX5 overexpression. Furthermore, we induced transient knockdown of OPN to reverse the tumorigenic behaviour of TSCC cells induced by IRX5 overexpression, which partially rescued the promoting effects of IRX5 on TSCC cell proliferation, migration and invasion.

The NF‐κB signalling pathway is involved in various physiologic and pathologic processes, including cancer initiation and promotion.^30^ It has been demonstrated that NF‐κB pathway is activated by OPN in cancers,^20^ we examined whether this pathway is activated by IRX5/OPN signalling. Our results revealed a positive correlation between IRX5 expression and NF‐κB pathway activity, and also demonstrated that BAY11‐7082 could partially reverse the IRX5‐induced TSCC biological behaviour. Moreover, knockdown of OPN expression in IRX5‐overexpressing cells led to a partial suppression of the NF‐κB pathway. These results confirmed that IRX5 activated the NF‐κB pathway by targeting OPN promoter. MMP2 acts as downstream of NF‐κB in cancers,^21^ where it degrades the ECM, which is a pivotal step in tumour metastasis and invasion.^31^, ^32^ In our study, MMP2 was up‐regulated by IRX5/OPN signalling, which partially explains the promoting effect of IRX5 in TSCC. In addition, NF‐κB is an important marker in high‐risk HNSCC patients and is, therefore, an attractive target for chemoprevention. However, because of several limitations, such as high cost, functional pleiotropy and safety concerns, NF‐κB inhibitors have not yet been used in clinical practice.^33^ We recommend the use of IRX5 as a novel molecular therapy target for TSCC.

In summary, our study demonstrated that IRX5 is up‐regulated in TSCC cells and tissues, which functionally enhances TSCC cells proliferation, migration and invasion in vitro as well as in xenograft tumour model in vivo. Mechanically, we identified that IRX5 overexpression expedites NF‐κB signalling pathway and ultimately promotes tumour growth. Notably, OPN is identified as a crucial target of IRX5, indicating that the IRX5/OPN/NF‐κB pathway is a novel mechanism of TSCC progression. Absolutely, direct evidence is required to provide a favourable novel biomarker and drug target for the diagnosis and treatment of TSCC.

## CONFLICT OF INTEREST

The authors confirm that there are no conflicts of interest.

## AUTHOR CONTRIBUTIONS

Liyuan Huang and Fangfang Song designed the experiments, performed experiments and analysed the data and also wrote the paper; Hualing Sun designed the experiments and provided study material; Lu Zhang provided study material; Cui Huang designed the experiments and provided study material and financial support. All authors read and approved the manuscript.
