# Supplementary material for: IRX5 promotes NF‐κB signalling to increase proliferation, migration and invasion via OPN in tongue squamous cell carcinoma
Source: J Cell Mol Med. 2018 May 15;22(8):3899–910. doi: 10.1111/jcmm.13664 (PMC6050492; doi:10.1111/jcmm.13664)
Supplement: Supplementary file 1 [file JCMM-22-3899-s001.docx]

Table S1. Primer sequences for qRT-PCR.

| Gene | Forward primer (5'-3') | Reverse primer (5'-3') |
| --- | --- | --- |
| IRX5  Cyclin D  GAPDH | GCAAAGACTCTCCCTATGA  AACTACCTGGACCGCTTCCT  TGAACGCTGCTTACATGCCA | CTCTCTCGGTGATGGAAA  CCACTTGAGCTTGTTCACCA  AGGCTGTTGTCATACTTCTC |
| OPN | CATCACCATCTTCCAGGAG | AGGCTGTTGTCATACTTCTC |
| OPN-promoter | CGGGGTACCtgacgcagcatccatttcct | CCGCTCGAGtcaagcctgcaaggagttca |
