# Supplementary material for: IRX5 promotes NF‐κB signalling to increase proliferation, migration and invasion via OPN in tongue squamous cell carcinoma
Source: J Cell Mol Med. 2018 May 15;22(8):3899–910. doi: 10.1111/jcmm.13664 (PMC6050492; doi:10.1111/jcmm.13664)
Supplement: Supplementary file 2 [file JCMM-22-3899-s002.docx]

Table S2. Sequences for siRNA.

| Gene | sense (5'-3') | antisense (5'-3') |
| --- | --- | --- |
| siIRX5#1  siIRX5#2  siNC | CAUCGUCGGACAAGGUCAATT  GAGAGAGACAGAGAGAGAATT  UUCUCCGAACGUGUCACGUTT | UUGACCUUGUCCGACGAUGTT  UUCUCUCUCUGUCUCUCUCTT  ACGUGACACGUUCGGAGAATT |
| siOPN#1  siOPN#2 | GUCUCACCAUUCUGAUGAATT  CCUGUGCCAUACCAGUUAATT | UUCAUCAGAAUGGUGAGACTT  UUAACUGGUAUGGCACAGGTT |
